# Obtaining Reliable Likelihood Ratio Tests from Simulated Likelihood Functions

**DOI:** 10.1371/journal.pone.0106136

**Published:** 2014-10-20

**Authors:** Laura Mørch Andersen

**Affiliations:** Department of Food and Resource Economics, University of Copenhagen, Frederiksberg, Copenhagen, Denmark; University of East Piedmont, Italy

## Abstract

**Mixed models:**

*Models allowing for continuous heterogeneity by assuming that value of one or more parameters follow a specified distribution have become increasingly popular. This is known as ‘mixing’ parameters, and it is standard practice by researchers - and the default option in many statistical programs - to base test statistics for mixed models on simulations using asymmetric draws (e.g. Halton draws).*

**Problem 1: Inconsistent LR tests due to asymmetric draws:**

*This paper shows that when the estimated likelihood functions depend on standard deviations of mixed parameters this practice is very likely to cause misleading test results for the number of draws usually used today. The paper illustrates that increasing the number of draws is a very inefficient solution strategy requiring very large numbers of draws to ensure against misleading test statistics. The main conclusion of this paper is that the problem can be solved completely by using fully antithetic draws, and that using one dimensionally antithetic draws is not enough to solve the problem.*

**Problem 2: Maintaining the correct dimensions when reducing the mixing distribution:**

*A second point of the paper is that even when fully antithetic draws are used, models reducing the dimension of the mixing distribution must replicate the relevant dimensions of the quasi-random draws in the simulation of the restricted likelihood. Again this is not standard in research or statistical programs. The paper therefore recommends using fully antithetic draws replicating the relevant dimensions of the quasi-random draws in the simulation of the restricted likelihood and that this should become the default option in statistical programs. JEL classification:* C15; C25.

## Introduction

Models allowing for continuous heterogeneity have been developing rapidly thanks to advances in computational speed and understanding of simulation methods for approximating integrals, (see e.g. [1]; [2]; [3]; [4]; [5] or [6]). One way of introducing continuous heterogeneity is to assume that value of one or more parameters follow a specified distribution. This is known as ‘mixing’ parameters, and the result of the estimation is the moments characterising the mixing distribution rather than a single value of the mixed parameter. Calculating the likelihood of a mixed model means that a conventional likelihood must be integrated over all possible values of the mixed parameters weighted by the mixing density. Often, this integral does not have a closed form and the integral is therefore approximated by either Monte Carlo or Quasi-Monte Carlo integration. This means that values of the mixed parameter are drawn either randomly or quasi-randomly from the underlying distribution and used to calculate the numerical integral which is then used as an approximation.

Many models are estimated by simulated maximum likelihood, and restrictions easily tested using Likelihood Ratio tests. As an example, the Mixed MultiNomial Logit (MMNL, [7]) is now a standard way for researchers to introduce continuous heterogeneity into discrete models, and Mixed MultiNomial Logit is now available in many different software packages. [8] compares the accuracy of Mixed MultiNomial Logit estimation in SAS, NLOGIT-LIMDEP and a user-written add-in module for Stata. They find it strange that both SAS and the Stata module allows the estimated standard deviations of the mixing distributions to be negative, and that both packages advice the user to reverse the sign in these cases. There is however nothing strange about the negative values of the estimated standard deviations of the mixing distributions. If the mixed distribution depends on the value of a variance, it is common to maximize the likelihood value over the standard deviation which is the square root of the variance. This relationship between the variance and the standard deviation means that the likelihood function will be symmetric around zero for the standard deviation, and the simulated likelihood must therefore also be symmetric around zero in this dimension. The statistical software Biogeme also sometimes report negative standard deviations, and the results of estimations using Biogeme vary depending on the sign of the starting values for the standard deviations. As will be illustrated in the following, this is problematic because standard deviations with identical absolute values should lead to the same likelihood value independent of the sign, and therefore also to the same result when maximizing the likelihood.

This paper illustrates that if the conventional likelihood is symmetric the simulated mixed likelihoods will always be symmetric, but if the conventional likelihood is asymmetric this is not true unless the draws for the Quasi-Monte Carlo integration are also symmetric around zero. Antithetic draws have been suggested as a variance reduction technique for Monte Carlo integration of asymmetric functions, especially for Bayesian inference (see e.g. [9] or [6]). However, the technique is not generally used in mixed models which also use Monte Carlo integration, and the simulated mixed likelihoods are therefore usually not symmetric. As the number of draws increases the degree of symmetry will increase, but as it will also be illustrated in the following, the degree of symmetry is not always sufficient within the range of draws usually applied. One example of an asymmetric conventional likelihood is the likelihood of a logit model, which will be used to illustrate the problem in this paper.

Antithetic Halton draws have also been suggested as an instrument for faster computation of Quasi-Monte Carlo integrals, allowing for more precise point estimates within a reasonable time frame (recently in [10]). In the present paper we focus on the reliability of Likelihood Ratio tests of mixed models instead of precision of point estimates or estimation speed. If Mixed MultiNomial Logit models are estimated without antithetic draws, Likelihood Ratio tests may be compromised, which again may lead to false conclusions. This paper illustrates why asymmetric draws are likely to lead to false Likelihood Ratio tests, and why antithetic draws solve this problem. The purpose of this paper is to support the idea of using fully antithetic draws for simulated likelihoods, and not least to warn researchers who use standard estimation procedures for e.g. Mixed MultiNomial Logit, that their Likelihood Ratio tests may be invalidated by the asymmetric nature of the draws.

Using data simulated under a Mixed MultiNomial Logit model specification, we illustrate how asymmetry of the simulated likelihood function causes the likelihood to depend on the signs of the estimated Choleski factorization, and that *the problem of inconsistent Likelihood Ratio tests caused by the asymmetry of the quasi-random draws is completely removed when one uses fully antithetic draws instead of conventional asymmetric draws.* Note that this solution only solves the problem of ‘false’ local maxima (where the parameters of the statistical model are uniquely identified, but the maximum likelihood function varies) which are caused by the asymmetry; it does NOT solve the problem of ‘true’ local maxima (where both likelihood value and parameter values vary) which can occur as a result of a non-linear utility function, or insufficient data. We also show that the same mechanisms appear in a real data set with invalidating implications for Likelihood Ratio tests.

Having solved the problem of inconsistent Likelihood Ratio tests caused by asymmetric draws, the paper turns to another problem which could still invalidate Likelihood Ratio tests, even when antithetic draws are used. When restricting the number of dimensions of the mixing distribution from *n* to *n*-1 most standard procedures simply estimate the restricted model using the *n*-1 first set of quasi-random draws, irrespective of which of the *n* original dimensions is restricted. As illustrated in this paper, keeping track of which dimension is restricted, and removing exactly this dimension when estimating the restricted model, may lead to better Likelihood Ratio tests.

The problem and solution presented in this paper not only applies to Mixed MultiNomial Logit models, but also to other models estimated by maximum simulated likelihood and the paper therefore provides a valuable contribution to the ongoing struggle to improve simulation methods. Halton draws are used in this paper, but the properties of the antithetic draws can be generalized to other types of draws.

The structure of the paper is: The above section introduced the problems associated with Likelihood Ratio tests performed on simulated log-likelihood values, if these are simulated using asymmetric draws. The following theory section consists of a subsection which outlines the standard way of estimating and testing within panel Mixed MultiNomial Logit models, a subsection which explains why asymmetric draws used in Quasi-Monte Carlo integration of mixed likelihoods may invalidate Likelihood Ratio tests, followed by two subsections which first illustrate the problem on simulated data by comparing results in the true optimum using conventional Halton draws and then using real data. The method section introduces antithetic Halton draws. The results section also contains two separate subsections, one which presents the encouraging results of using fully antithetic of draws and another which introduces the second problem which concerns tests reducing the dimension of the mixing distribution. The final section concludes on both problems.

## Theory

As mentioned above this section consists of four subsections, which introduces the problem of inconsistent likelihood ration tests due to asymmetric draws.

### 1. Estimation and testing in Panel Mixed Logit Models

In a conventional logit ([11]) it is assumed that all individuals have the same utility function, but in a Mixed MultiNomial Logit (MMNL or MXL) or Random Parameter Logit (RPL) model ([7]), it is assumed that (part of) the individual utility is drawn from a distribution. This means that the individual utility is known to the individual, but only the distribution is assumed to be observable to the econometrician. The mixed likelihood function is then the likelihood function of the conventional multinomial logit model integrated over all possible values of 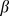, which in a panel mixed logit becomes ([12]):
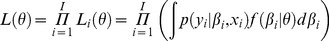
(1)where 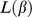 is the likelihood of the mixed logit given the mixing distribution of 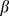 given by 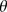, 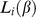 is the likelihood for individual *i*, *I* is the number of individuals, 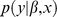 is the likelihood of a conventional logit model given 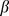 and 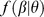 is the density of 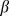 given *θ*. The likelihood function is maximized over *θ*.

Calculating the likelihood function in equation (1) is very cumbersome, especially if 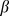 follows a multivariate distribution, but the problem can be reduced significantly by simulating the likelihood using either random draws (Monte-Carlo integration) or quasi-random draws (Quasi-Monte Carlo integration). In 2006, [13] suggested an algorithm using random draws, but with an execution time which was competitive with existing tools using Quasi-Monte Carlo for mixed logit models, but in general Quasi-Monte Carlo integration is found to be more efficient (see e.g. [19] for asymptotic properties of Quasi-Monte Carlo integration). This is often done using quasi-random Halton sequences which were first presented by [14] and [15]. The efficiency of Halton sequences compared to random draws is discussed in detail in both [16] and [17]. Both find that Halton sequences greatly improve accuracy, allowing for far fewer draws and faster computation. However, [18] finds that the coverage of standard Halton draws rapidly deteriorates as the dimension of the mixing distribution increases, and therefore suggests using scrambled Halton draws whenever the dimension exceeds five.

Clearly, reliable estimation, validation and inference techniques are a prerequisite for sound models and analysis. The use of simulated likelihoods are bound to induce some approximation error, and it is therefore important to validate the results, e.g. by varying the starting values of the parameters and checking the stability of the results. One example of a simulation error is investigated in [20], who illustrated that a low number of draws in the simulation of the integral may lead to unidentified estimates. [2], [21] and [22] confirm that in general the validity of the results is greatly influenced by the number of draws, which should therefore also be varied.

Many other types of quasi-random draws have been suggested and found to be more efficient than Halton draws, e.g. (t,m,s)-nets [23], scrambled Faure sequences [24] or randomly shifted Lattice cubes [25]. Just like the Halton draws, these other types are asymmetric, and the need for antithetic draws presented in this paper will most likely also apply to these other types. Halton draws are used as an example in this paper, as it is widely used in standard estimation programs. When optimizing a mixed likelihood function by Quasi-Monte Carlo integration the optimization routine uses the same set of quasi random draws for all potential values of the moments of the mixing distribution. This is done in order to ensure that the simulated likelihood values for different values of moments are as comparable as possible. As shall be illustrated in the following, the property of identical draws is in some cases violated if the quasi random draws are asymmetric, and as will also be illustrated in the following, this may have serious consequences for the inference of the estimated models.

In many cases the purpose of estimating a likelihood function is twofold: Maximizing the likelihood function leads to the set of parameters which fit the data best, and comparing the best likelihood values of different models makes it possible to infer whether the models are significantly different. The latter is done by Likelihood Ratio (LR) tests based on the difference between the restricted and the unrestricted likelihood values (see e.g. [26]). If the underlying utility function is linear, the theoretical statistical model has one and only one maximum log-likelihood value, but in some cases the value of the simulated maximum log-likelihood varies with the starting values. This phenomenon is usually ascribed to local maxima, which may occur if the variation in data is not large enough to support the statistical model, or if the underlying utility function is non-linear and allows for multiple maxima. If local maxima is the problem both the value of the likelihood function and the estimated parameters will vary with the starting values, and the problem can sometimes be recognized by varying the starting values.

This paper however point to a very different and more serious type of variation in the value of the maximum log-likelihood. We find that the use of asymmetric draws for Quasi-Monte Carlo simulation of mixed likelihoods may lead to cases where the parameters of the statistical model are uniquely identified, but the maximum likelihood function varies, we call this ‘false’ local maxima, whereas ‘true’ local maxima varies both in likelihood value and parameter value. The absolute level of the log-likelihood function is of no interest, but if the difference in the value of the simulated log-likelihood given different starting values of the parameters is above e.g. two, testing hypotheses may easily lead to false conclusions. For a Chi-square distribution with one degree of freedom the critical value is 3.84 at the 5% level. This means that if the difference between the unrestricted and the restricted log-likelihood is above 1.92 (half of 3.84) the null hypothesis will be rejected at the five percent level in a test with one degree of freedom. The varying values of the log-likelihood function may first of all lead to falsely accepted or rejected hypotheses. Secondly, it may also falsely indicate that data are not informative enough to support the model, and therefore lead to unnecessary reductions in model complexity.

### 2. The effect of asymmetric draws on simulated log-likelihood values

If one parameter in a conventional logit model is mixed with e.g. the normal distribution, two moments characterizing the distribution are estimated, a mean and a standard deviation. When estimating the mean and the standard deviation of the mixing distribution, these are usually both maximized over the entire real axis 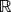. Actually, the mixing distribution depends on the *variance* rather than the standard deviation, and since the variance is the square of the standard deviation the true mixed likelihood function of the standard deviation will be symmetric around zero.

In most cases however, the true value of the likelihood value of a Mixed MultiNomial Logit model is not possible to obtain, and instead the value is often simulated using Quasi-Monte Carlo integration. This means that the value of the likelihood function of the conventional likelihood is calculated for a number of quasi-randomly drawn 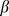
’s and the average of these values is used as an approximation of the integral in equation (1). Formally the likelihood function for the entire sample becomes:
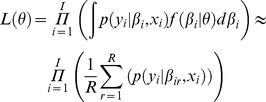
(2)where *R* is the number of draws and the 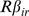
’s are drawn quasi-randomly from the mixing distribution given by 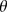.

We choose to use the normal distribution as an example of a mixing distribution, which means that if the mixing distribution is one dimensional then for each individual *i*, the *r* ^th^ draw is created as
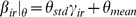
(3)where 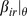 is 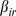 given 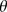, *θ_std_* is the standard deviation of the mixing distribution, 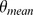 is the mean of the mixing distribution and 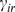 is a quasi-random draw from a standard normal distribution with mean zero and standard deviation one. In the following we will investigate the effect of symmetry versus asymmetry of the draws 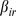 around the mean 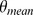, and for simplicity 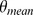 is therefore set to zero. When 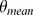 is zero, 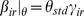 for all *i* and all *r*, and therefore:
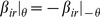
(4)where 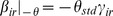.

If the conventional likelihood *p* is symmetric in 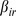 then 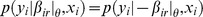, which because of (4) also means that 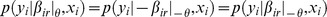 for all values of *i*, *r* and 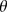, and therefore that the Quasi-Monte Carlo integral in equation (2) is independent of the sign of the standard deviation. If *p* is not symmetric in 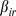, then the Quasi-Monte Carlo integral is only identical for 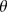 and 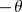 if for every combination of *i* and *r* there exists a value *s* so that 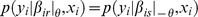. If the quasi-random draws 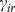 are symmetric around zero then this is always the case, but if the quasi-random draws are not symmetric around zero then 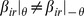, at least for some combinations of *i*’s and *r*’s.

This means that the simulated mixed likelihood of *θ* is not identical to the simulated mixed likelihood of 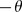 
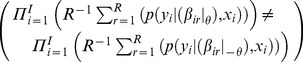
 when an asymmetric likelihood is mixed using asymmetric draws, because the draws are not identical on the positive and the negative half of the real axis 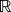, and the results using 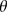 or 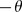 therefore are incomparable. The sign of the standard deviation therefore influences the value of the simulated mixed likelihood, even though the sign has no influence on the variance of the mixing distribution. Clearly, the problem decreases as the number of draws increases, and the symmetry of the draws therefore increases, and the important question is therefore whether the variation in likelihood values caused by asymmetric draws poses a real problem within the range of draws usually used today. As will be illustrated in the following sections, this is unfortunately the case.

If the mixing distribution is multivariate, the draws are created as 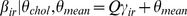 where *Q* is the triangular Choleski factorization and 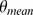 is a vector of means. The Choleski factorization (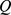) is a triangular matrix with the property *QQ’* = 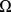, where 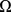 is the variance-covariance matrix [27]. If the variance-covariance matrix is diagonal (i.e. no correlations) the Choleski matrix is merely a diagonal matrix of standard deviations In the case of an *n*-dimensional mixing, the variance-covariance matrix may be obtained by 2*^n^* different Choleski factorizations with different combinations of signs of the elements of the triangular matrix. This means that if an asymmetric likelihood function such as the conventional logit model is mixed with a distribution which depends on a Choleski factorization of a variance-covariance matrix, using asymmetric draws, the combination of signs of the estimated Choleski factorization will wary between quadrants, but all lead to the same variance-covariance matrix. The values of the parameter estimates are therefore not affected by the lack of symmetry of the quasi-random draws, but the optimal value of the mixed likelihood may differ between the 2*^n^* quadrants. As shall be illustrated in the following section, this problem can in some cases invalidate Likelihood Ratio tests.

### 3. Illustrating the problem using simulated data

In order to investigate the magnitude of the problem under controlled conditions, a hypothetical data set has been simulated. The data are panel data with 1,000 individuals each making 20 choices between 4 alternatives. The utility of the alternative specific constant is zero for the alternative which is used as base; the utility of the remaining alternatives follows a three-dimensional normal distribution with no correlation and nonzero means. In the case of three mixings, the variance-covariance matrix is estimated in 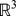, which means that the number of different quadrants is 2^3^ = 8, and the likelihood function must therefore be symmetric in all eight quadrants.

One of the virtues of simulated data is that the true mean and variance-covariance of the mixing distribution are known. The finite nature of the simulated data means that the true (realized) means and standard deviations of utility in the sample are not identical with the values used in the simulation of the data but in the rest of this section, the likelihood values will be evaluated in the true mean and variance-covariance of the simulated data for each of the eight quadrants, and compared between the different quadrants. The purpose of this section is to illustrate the potential problem using simulated data, ignoring the optimization errors caused by different optimization routines. We use therefore use the term ‘evaluated’ rather than ‘estimated’ because the likelihood function is not *optimized* over each quadrant, but instead *calculated* by Quasi-Monte Carlo integration in the optimal point which is known because the data is simulated. The evaluation is done by Quasi-Monte Carlo integration, using standard Halton draws. In these calculations, the probability of finding an optimum in a given quadrant is treated as equal for all quadrants. In actual estimations the probability of ending up in a given quadrant may well vary, and the results in this section therefore only illustrate the *magnitude* of the problems that may *potentially* arise from actual estimations. As will be illustrated in section 0, estimations on actual data lead to results in all eight quadrants, so the problem also exists when the modes of the distribution of the mixed parameters are optimized rather than known a priory.

Table 1 compares the results from the eight quadrants for increasing numbers of draws. All calculations on simulated data are conducted in the MMNL GAUSS program developed by Train, Revelt and Ruud (see acknowledgements). The antithetic Haltons are added to the program by the author of the present paper. The Choleski factorization of the variance-covariance matrix of the mixing distribution in the different quadrants vary by the combination of signs of the Choleski factorization, but all leads to the same (true) variance-covariance matrix and all have the same (true) means. For 100 draws, the highest difference between the log-likelihood values of different quadrants is 9.26 which is definitely not zero as it should theoretically be. As mentioned above, differences of this magnitude can ruin Likelihood Ratio tests completely. The difference between the quadrants decreases as the number of draws increases, simply because the distance between draws is reduced, but it does not disappear within a feasible span of draws. Table 1 shows the results in the true optimum, but the difference in likelihood values between quadrants is even higher outside the optimum. This problem decreases to some extent with the number of draws, but as illustrated in [28], the difference still does not disappear even with 7,500 draws, and the problem is sometimes smaller for 5,000 draws than for 7,500 draws. This has to do with the degree of symmetry of the Halton draws. As illustrated in [28], the degree of symmetry increases as the number of draws increases, but not monotonically. Increasing the number of draws by a few thousand may therefore lead to set of draws with a lower degree of symmetry, and thereby a bigger difference between the likelihood values in different quadrants. This might be the reason why [27], (page 230) find lower standard deviations of the estimated parameters using 100 Halton draws compared to 1,000 Halton draws. This is not explored in the present paper, but would be interesting to investigate further in the future.

**Table 1:** Variation in simulated log-likelihood, simulated data, conventional Halton draws.

	Number of draws per individual					
	100	500	1,000	1,500	5,000	7,500
Highest absolute difference in simulated log-likelihood,evaluated in true means and true variance-covariance matrixin different quadrants	9.26	4.44	1.09	0.88	0.47	0.19

Simulated log-likelihood in a model with 4 alternatives and 3 mixed alternative specific constants. Simulated data, 1,000 individuals, 20 observations per individual. Data is defined in S1: Simulated data.

As mentioned above, one of the problems caused by the difference between the values of the log-likelihood function evaluated at different quadrants is that it influences the results of Likelihood Ratio tests. The rest of this section investigates the effect on a Likelihood Ratio test of the null hypothesis that the mean utility of alternative B is zero. Note that the base alternative A has both mean *and* standard deviation equal to zero. Testing whether the mean of alternative B is equal to zero is therefore *not* the same as testing whether the utility of alternative B is the same as the utility of the base alternative A.

**Figure 1:**
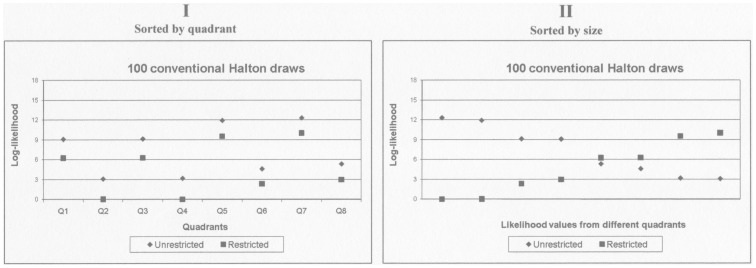
Differences between log-likelihood values of unrestricted and restricted model. The null hypothesis is that the mean utility of alternative B is zero. Panel **I** shows the values sorted by quadrant. Panel **II** shows the values sorted by size.

The large variation in the value of the log-likelihood function means that the value of the restricted model in one quadrant may be higher than the value of the unrestricted model in another quadrant, but never within quadrants. Figure 1 shows the simulated log-likelihood values for the unrestricted and the restricted model using 100 Halton draws. The model is presented in the supporting information file ‘S1: Simulated data’, and has 4 alternatives and 3 mixed alternative specific constants. Only the difference between likelihoods is interesting, and the lowest estimated log-likelihood value (from the restricted model in Q4) is therefore subtracted from all the estimated values.

**I** shows the relationship between the two models in each quadrant and **II** ignores the quadrants and sorts the eight values by size. Especially from **II** it is evident that the value of the restricted model will sometimes be higher than the value of the unrestricted model, leading to negative values of the LR test statistic.

**Figure 2:**
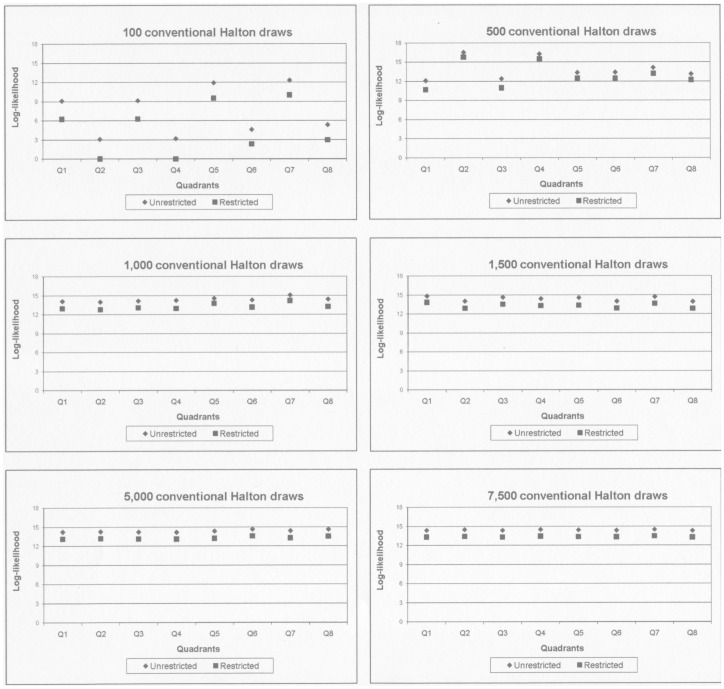
Log-likelihood function evaluated in the true parameter values of the simulated data, by quadrant. Simulated data, 1,000 individuals, 20 observations per individual. Data is defined in S1: Simulated data. Calculations conducted in the MMNL GAUSS program developed by Train, Revelt and Ruud, using conventional Halton draws. The null hypothesis is that the mean utility of alternative B is zero.

**Figure 3:**
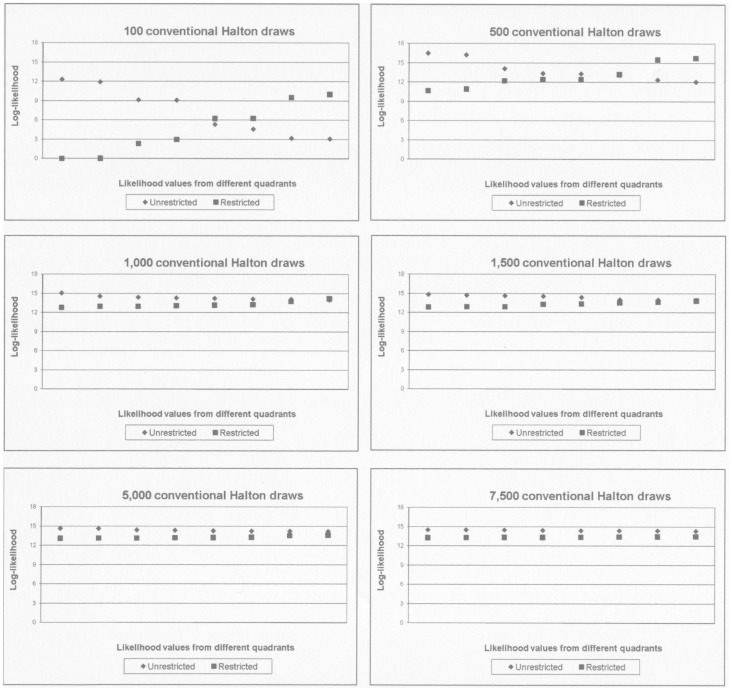
Log-likelihood function evaluated in the true parameter values of the simulated data, by size. Simulated data, 1,000 individuals, 20 observations per individual. Data is defined in S1: Simulated data. Calculations conducted in the MMNL GAUSS program developed by Train, Revelt and Ruud, using conventional Halton draws. The null hypothesis is that the mean utility of alternative B is zero.

Table 2 shows that for 100 conventional Halton draws, the LR test statistic will become negative in 20 of the 64 different combinations of restricted and unrestricted log-likelihood values, corresponding to 31 per cent of the cases. The problem decreases with the number of draws, but is still present at 1,000 draws. Figure 2 and Figure 3 repeats Figure 1 for all the different numbers of draws presented in Table 2.

**Table 2:** Restricting one mean using conventional Halton draws.

	Number of draws per individual:					
	100	500	1,000	1,500	5,000	7,500
Share of negative LR values	.31	.31	.05	.00	.00	.00
*Results of positive LR values*						
Lowest p-value	.00	.00	.03	.05	.08	.12
Highest p-value	.64	.66	.77	.65	.27	.20
Standard deviation ofp-values^a^	.13	.16	.15	.13	.05	.02

Data is defined in S1: Simulated data. The null hypothesis is that the mean utility of alternative B is zero. a: Standard deviation calculated from the p-values of the positive LR values from the 64 different combinations of quadrants.

Table 2 also summarizes the results of the Likelihood Ratio tests that can be performed on the positive LR test statistics. The p-values vary from zero to 64 per cent for 100 draws leading to a standard deviation of 13 per cent. Note that these tests are all performed on the *same* data set. Had the test been performed on different realizations of data with identical values of the mean and the variance-covariance, the test should have been accepted on 10 per cent of the data sets at the ten percent level, but when the tests are performed at the same dataset the results should all be identical. The differences are caused by the asymmetry of the Halton draws used in the Quasi-Monte Carlo integration of the likelihood, not by statistical properties of the test.

The standard deviation of the p-values presented in Table 2 is of course deeply problematic, because it means that the result of the Likelihood Ratio test is likely to be unreliable. Table 3 shows the probability of rejecting the null hypothesis at different significance levels. Using 100 draws, 55 per cent of the positive combinations of unrestricted and restricted log-likelihood values reject the null hypothesis at the 1 per cent significance level and in 91 per cent of the cases the null hypothesis is rejected at the 10 per cent level. Using 7,500 draws, the model is never rejected. Table 3 therefore shows that the problem decreases as the number of draws increases but even for 5,000 draws the null hypothesis will sometimes be accepted at the 10 per cent level, and other times rejected.

**Table 3:** Probability of rejecting the null hypothesis.

	Number of draws per individual:					
	100	500	1,000	1,500	5,000	7,500
At the 1 per cent level	.55	.25	.00	.00	.00	.00
At the 5 per cent level	.89	.45	.07	.05	.00	.00
At the 10 per cent level	.91	.59	.28	.30	.17	.00

Data is defined in S1: Simulated data. The null hypothesis is that the mean utility of alternative B is zero.

### 4. An example using real data

The problem described above has also been experienced on real data. The example below is based on 10,971 observations from 848 individuals, choosing between four different alternatives. The data has been used to estimate willingness to pay for eggs with different levels of animal welfare (see [29] where antithetic draws are used in the estimation). The utility of the non-base alternatives is assumed to follow a three-dimensional normal distribution with correlation. In this example the true values of the means and variance-covariance of the mixing distribution are not known, and the model is therefore *optimized* using 52 different sets of starting values. All calculations on real data are conducted in an extension of the MMNL GAUSS program developed by Train, Revelt and Ruud, which allows for correlation between mixed parameters. The antithetic Haltons are added to the program by the author of the present paper.

The optimized log-likelihood values of 52 different sets of starting values have been sorted into quadrants by the sign of the estimated Choleski factorization, and table 4 shows the optimized values of the log-likelihood function in the eight different quadrants, along with the probability of finding a maximum in each quadrant. The estimated simulated log-likelihood values differ significantly between the eight quadrants, but except for one value in Q2 they are identical within quadrants for a given number of draws (not shown). Comparing the quadrants of the starting values and of the optimized results shows that there is apparently no connection between the quadrant of the starting point and quadrant of the final result (not shown), so the quadrant of the estimation results cannot be influenced a priori.

**Table 4:** Maximum simulated log-likelihood values by quadrant. Real data, estimated optima, conventional Halton draws.

	Number of draws per individual:							
	1,000		1,500		5,000		10,000	
	Prob. ofquadrant*	log-like-lihood**	Prob. ofquadrant	log-like-lihood	Prob. ofquadrant	log-like-lihood	Prob. ofquadrant	log-like-lihood
Q1	19%	10.69	23%	7.83	17%	11.88	15%	10.23
Q2	8%	0.00***	12%	8.64	8%	11.98	15%	12.58
Q3	25%	6.64	6%	11.27	17%	11.79	13%	11.45
Q4	19%	8.72	8%	11.32	23%	10.55	21%	11.19
Q5	15%	5.24	19%	11.90	12%	12.80	13%	11.64
Q5	6%	7.05	15%	13.05	6%	12.03	6%	10.99
Q7	4%	4.29	8%	13.76	10%	12.24	13%	12.51
Q8	4%	10.88	10%	13.85	8%	11.37	2%	11.43
*Largest difference*		*10.88*		*6.02*		*2.26*		*2.35*

*This is the probability that the estimated Choleski factorization lies in this quadrant. The estimation is performed 52 times with different sets of starting values.

**Only the difference between likelihoods is interesting, and the lowest estimated log-likelihood value (−8,388.20, from the estimation with 1,000 draws in Q2) is therefore subtracted from all the estimated values, in order to make the table easier to read.

***One of the four results in this quadrant differs from the others by 0.274.

The optimized log-likelihood values of 52 different sets of starting values have been sorted into quadrants by the sign of the estimated Choleski factorization, and table 4 shows the optimized values of the log-likelihood function in the eight different quadrants, along with the probability of finding a maximum in each quadrant. The estimated simulated log-likelihood values differ significantly between the eight quadrants, but except for one value in Q2 they are identical within quadrants for a given number of draws (not shown). Comparing the quadrants of the starting values and of the optimized results shows that there is apparently no connection between the quadrant of the starting point and quadrant of the final result (not shown), so the quadrant of the estimation results cannot be influenced a priori.

Table 4 shows that the difference between the quadrants is smaller for 5,000 draws than for 1,500 draws. The problem thus decreases with the number of draws, but even with 10,000 draws the problem is still present, as the maximized simulated log-likelihood still varies by 2.35 between quadrants, a difference which is large enough to invalidate Likelihood Ratio tests. As mentioned in the second section, a likehood Ratio test with one degree of freedom levels leads to a rejection of the null hypothesis if the difference between the unrestricted and the restricted log-likelihood is 1.92 or higher. The problem of unreliable Likelihood Ratio tests which was illustrated using simulated data in the previous section therefore also appears on real data. In this example, the variation in simulated likelihood values is sufficiently large to invalidate Likelihood Ratio tests, even for high numbers of draws. It is important to note that whereas the log-likelihood values vary problematically much between the quadrants of the Choleski factorization, the resulting variance-covariance matrices are identical between quadrants, so the problem only affects Likelihood Ratio tests, not the validity of the estimated parameters.

## Method

This section introduces the concept of Antithetic Halton draws. If the model includes more than one mixed parameter, symmetry in one dimension is not enough. If the number of mixed parameters is *n* – and if perfect symmetry is the goal – for each point in a given quadrant a corresponding point must be present in all of the other 2*^n^*-1 quadrants. The problem is solved by creating antithetic Halton draws. As in [27], the draws are created so that each point is ‘mirrored’ into the 2*^n^*-1 other dimensions. We call these draws fully antithetic because they are antithetic in all dimensions.

For a case with three mixed parameters a Halton draw 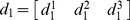 (each between zero and one) is drawn, and then paired with 7 mirrors in the following way:
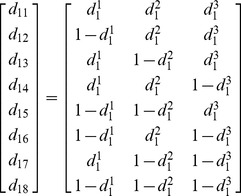
(6)


The Haltons must be symmetric for each individual in the panel, and it is therefore important that each ‘set’ of symmetric draws is assigned to one individual only, and not distributed over different individuals. The number of draws per individual in a model with *n*-dimensional mixing must therefore be a multiple of 2*^n^*. In the case of 1,500 draws and three mixings this means that the number of draws must be e.g. 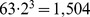 instead of 1,500 to ensure symmetry. Antithetic draws always have perfect symmetry, and therefore always skewness coefficient equal to zero (see e.g. [26], for a definition of the skewness coefficient). [30] provide Stata programs for calculating multivariate normal probabilities by simulation, and allow for antithetic Halton draws. However, the antithetic draws are only one dimensionally antithetic, ignoring the 2*^n^* ^-1^ other dimensions. This means that these draws will still allow for 2*^n^* ^-1^ different local maxima, and that this type of one dimensionally antithetic draws therefore do not solve the problem of unreliable Likelihood Ratio tests. The following section shows that the fully antithetic draws described above solve the problem of simulation driven local maxima, and therefore provides reliable Likelihood Ratio tests.

## Results

This section has two subsections. One which presents the results of antithetic Halton draws, and one which present the second problem dealt with in this paper, the problem of maintaining the correct mixing dimensions when reducing the number of mixed parameters.

### 1. Results of antithetic Halton draws

When the simulated likelihood function for the simulated data is calculated using antithetic Halton draws, the difference between the log-likelihood values from different quadrants is always zero as desired, and the Likelihood Ratio test of the null hypothesis therefore no longer varies. However, the result still changes as the number of draws increases. Table 5 presents the p-values for the simulated data which were also presented in Table 2 above, combined with the results of the antithetic draws.

**Table 5:** Restricting one mean using conventional or antithetic Halton draws, simulated data.

	Number of draws per individual:					
	100	500	1,000	1,500	5,000	7,500
*Conventional Halton draws:* *(as in * *Table 2* *)*						
Lowest p-value	.00	.00	.03	.05	.08	.12
Highest p-value	.64	.66	.77	.65	.27	.20
*Antithetic Halton draws:*						
Lowest and highest p-value	.01	.08	.11	.09	.15	.15

Data is defined in S1: Simulated data. The null hypothesis is that the mean utility of alternative B is zero.

Table 6 compares the differences in log-likelihood values on real data presented in Table 4 with the results of antithetic draws, and clearly demonstrates that the antithetic Halton draws provide a more efficient way of achieving the correct result. The precision of the optimization is set to 10^−4^, and the highest difference between two results using antithetic draws is now lower than twice this level, and thereby completely acceptable. The precision of the optimization indicates how close to zero the gradient of the log-likelihood function must be to be perceived as a maximum. Differences of this magnitude will have absolutely no effect on Likelihood Ratio tests, and the antithetic Halton draws therefore solve the problem of ’false’ local maxima of the simulated likelihood function which will occur if both the likelihood function and the draws are asymmetric. Note, however, that local maxima may still occur if the utility function is non-linear, or if the model cannot be empirically identified by the data. The stability of the simulated log-likelihood should therefore still be investigated by estimations using different sets of starting values of the parameters, and standard estimation procedures should therefore also allow the user to control the starting values.

**Table 6:** Difference in simulated log-likelihood between quadrants, by number of draws, real data, conventional or antithetic Halton draws.

	Number of draws per individual			
	1,000	1,500	5,000	10,000
*Conventional Haltons (as in * *Table 4* *)*				
Highest absolute difference in simulated log-likelihood	10.88	6.02	2.26	2.35
*Antithetic Haltons*				
Highest absolute difference in simulated log-likelihood	0.000196			

### 2. Reducing the dimension of the mixing distribution

Even when the problem of symmetry is solved by using full dimensionally antithetic draws, the problem of comparing log-likelihood values of models with different dimensions still remains. In a model with two mixed parameters (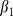 and 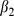) the Halton draws will be based on two primes, e.g. 2 and 3 (2 representing 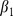 and 3 representing 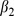). If one of the mixed parameters (e.g. 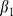) is restricted to be fixed (standard deviation restricted to zero), the dimension of the log-likelihood function is decreased by one, and the Halton draws will be based on only one prime. The standard choice would be the first prime, i.e. 2, independent of which dimension is restricted.

**Figure 4:**
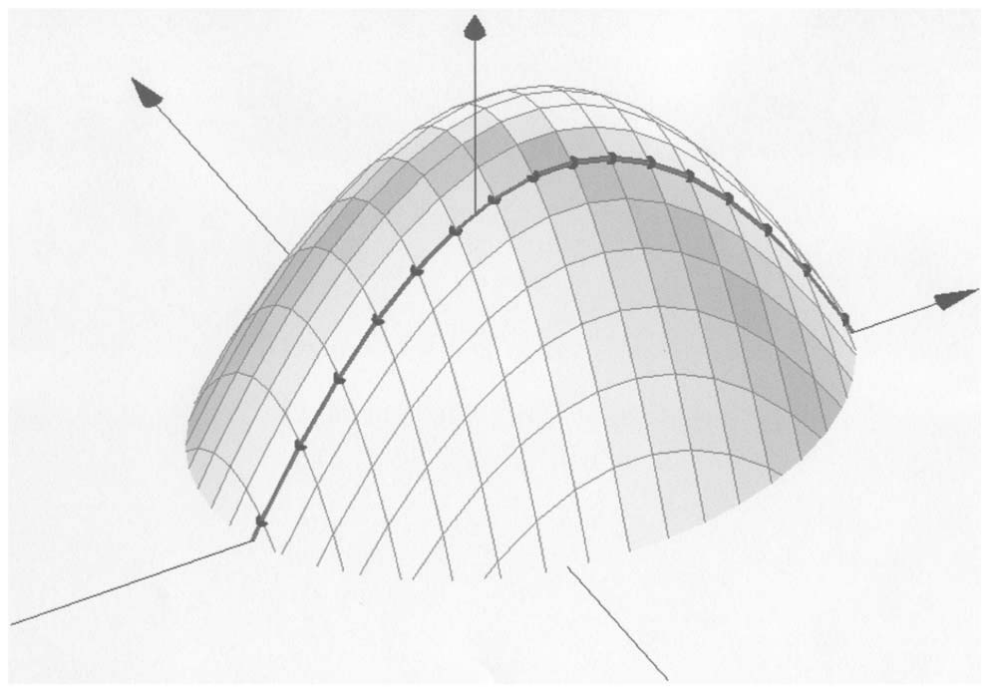
Simulated log-likelihood function in one and two dimensions. The figure describes a hypothetical log-likelihood function on a two dimensional parameter space. The heavy black line shows the one dimensional likelihood function when one of the standard deviation parameters is restricted to zero.

**Figure 5:**
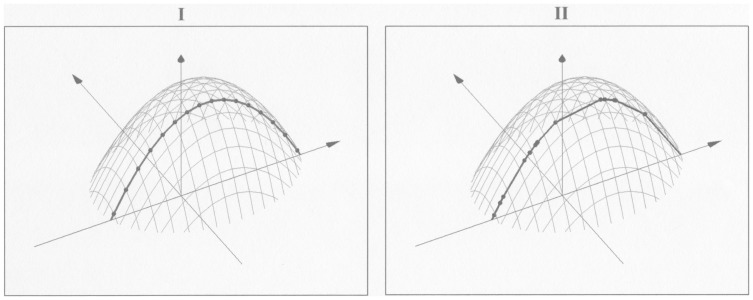
Different one-dimensional likelihood functions given by different draws. The figure describes the same hypothetical log-likelihood function as in figure 2. Panel **I** illustrates a case where the one-dimensional draws correspond with the two-dimensional grid. Panel **II** illustrates a case where the one-dimensional draws are not part of the two-dimensional grid.

Figure 4 illustrates the simulated conventional likelihood function which is to be integrated to form the likelihood function of the mixed logit. The heavy black line shows the likelihood function when one of the parameters is restricted to zero, and the dots on this line show the points in which the one-dimensional log-likelihood function would be evaluated for the given grid. The symmetry of antithetic Haltons is needed to ensure that the log-likelihood functions of the different quadrants are identical, but as illustrated in figure 5, the choice of prime may also matter.

Figure 5 describes the same hypothetical log-likelihood function as Figure 4. The dots show the points in which the one-dimensional log-likelihood function would be evaluated for different draws. The dots in **I** illustrate a case where the one-dimensional draws correspond with the two-dimensional grid, and **II** illustrates a case where the one-dimensional draws are not part of the two-dimensional grid. The area under the one-dimensional likelihood function is clearly not the same in **I** and **II**.

To investigate the size of the problem we return to the three dimensional mixing on the simulated data used above (1,000 individuals and 20 observations per individual, defined in S1: Simulated data). The restriction is now placed on the standard deviation of the utility of alternative C instead of the mean utility of alternative B, which was restricted in the mean-restriction case above. The utility of alternative C has a mean of 0.9981 and a standard deviation of 0.0984. The restricted model in this example assumes that the standard deviation is zero, but places no bounds on the mean. This means that the restricted model does not assume that the utility of alternative C is the same as the utility of the base alternative A. Table 7 shows the results of evaluating the log-likelihood function in the true parameters of the restricted model, using different primes for the antithetic Haltons. The differences may seem small compared to the absolute values of the likelihood functions, but remember that when restricting a single parameter, a difference of 1.92 between the restricted and the unrestricted likelihood will lead to acceptance of the restricted model at the five percent level. The difference between likelihoods based on different primes is therefore substantial, and for Likelihood Ratio tests which are ‘close’ to being accepted it will be important to keep track of the relationships between primes and mixing dimensions.

**Table 7:** Log-likelihood values in the optimum of the restricted model using antithetic Haltons.

	Number of draws per individual:					
	100	500	1,000	1,500	5,000	7,500
Antith. Haltons based on 2 and 3	−18,466	−18,413	−18,412	−18,410	−18,409	−18,408
Antith. Haltons based on 2 and 5	−18,475	−18,415	−18,410	−18,411	−18,409	−18,408
Difference between resultsbased on different primes	8.37	2.98	−1.64	0.92	−0.70	−0.85

The restricted model assumes that the standard deviation of the utility of alternative C is zero.

## Conclusion

This paper presents two problems which both mean that Likelihood Ratio test in mixed models estimated by standard methods cannot always be trusted. The first problem is that when a model with *n*-dimensional normal mixing is estimated by Quasi-Monte Carlo integration using asymmetric draws, the estimated variance-covariance matrix may be obtained from 2*^n^* different Choleski factorizations, located in different quadrants of the 2*^n^* dimensional parameter space, and these different factorizations may lead to different values of the optimized log-likelihood, even though they all lead to the same variance-covariance matrix. The paper shows that if the solution to an unrestricted and a restricted model is found in different quadrants, the Likelihood Ratio test is not reliable, and the paper also demonstrates that using fully antithetic draws eliminates this problem. The problem could also be solved by increasing the number of draws; but fully antithetic draws allows for faster computation and not least provides more reliable results for a given number of draws.

Halton draws and the normal distribution are used in this paper, but the need for fully antithetic draws will most likely also be relevant for other types of asymmetric draws, and for other types of distributions. As presented earlier, many other types of draws are used, but just like the Halton draws these are asymmetric. This paper shows that whenever the theoretical utility function is symmetric in one or more parameters (as for the Choleski factorisation of the normal distribution) the draws used for Quasi-Monte Carlo simulation must also be symmetric. Other distributions - such as the triangular distribution and the log-normal distribution - also have parameters which can only be positive, and are therefore likely to suffer from the same problem as the normal distribution. Antithetic draws are therefore strongly recommended for future use in standard estimation procedures for mixed models.

Some estimation procedures allow for one-dimensionally antithetic draws, but as explained above, one-dimensionally antithetic draws only removes one dimension of the problem, leaving 2*^n^* ^-1^ different optima which still may lead to false Likelihood Ratio tests. In the three dimensional mixings used in this paper, the dimension of the full set of antithetic draws is only eight and it is therefore possible to use fully antithetic draws, but in cases of higher dimensional mixings this may not be possible. In many applications the dimension of the mixing distribution will be small enough to use fully antithetic draws, and in these cases fully antithetic draws will ensure reliable Likelihood Ratio tests. Fully antithetic draws are therefore recommended for ‘small’-dimensional mixings.

The second problem illustrated in this paper is that even when fully antithetic Halton draws are used, testing restrictions on the number of mixed parameters may lead to false inference if the relationship between primes (which are the base of Halton draws) and mixed parameters are not maintained in the restricted model. Again, the problem could also be solved by increasing the number of draws; but keeping track of base values for different dimensions allows for faster computation and not least provides more reliable results for a given number of draws.

We therefore also recommend that for all types of quasi random draws, the user should be able to assign specific base values to specific dimensions, so that the set of base values can be maintained even when reducing the number of dimensions.

## Supporting Information

Appendix S1 (DOC)

## References

[1] Ben-Akiva M, Bolduc D, Bradley M (1993) Estimation of travel choice models with randomly distributed values of time. Transportation Research Record 1413: 88–97.

[2] Ben-Akiva M, Bolduc D (1996) Multinomial probit with a logit kernel and a general parametric specification of the covariance structure. Working paper, Department of Civil Engineering, Massachusetts Institute of Technology.

[3] Berry S, Levinsohn J, Pakes A (1995) Automobile Prices in Market Equilibrium. Econometrica 63: 841–890.

[4] Bhat CR (1996) Accommodating variations in responsiveness to level-of-service measures in travel model choice modelling. Working paper, Department of Civil Engineering, University of Massachusetts at Amherst.

[5] Brownstone D, Train K (1999) Forecasting new product penetration with flexible substitution patterns. Journal of Econometrics 89: 109–129.

[6] Geweke J (1996) Monte Carlo Simulation and Numerical Integration, in Handbook of Computational Economics, Volume L Edited by H.M. Amman, D.A. Kendrick and J. Rust.

[7] McFadden D, Train K (2000) Mixed MNL models for discrete response. Journal of Applied Econometrics 15: 447–470.

[8] Chang JB, Lusk JL (2011) Mixed logit models: Accuracy and software choice. Software review in Journal of Applied Econometrics 26: 167–172.

[9] Geweke J (1988) Antithetic Acceleration of Monte Carlo Integration in Bayesian Inference. Journal of Econometrics 38: 73–89.

[10] Sidharthan R, Srinivasan KK (2010) Random Coefficient Mixed Logit Models Based on Generalized Antithetic Halton Draws and Double Base Shuffling. Transportation Research Record: Journal of the Transportation Research Board 2175: 1–9.

[11] McFadden D (1973) Conditional logit analysis of qualitative choice behaviour. In P. Zarembka (ed.): Frontiers in econometrics. Academic Press, New York. USA, 105–142.

[12] Revelt D, Train K (1998) Mixed logit with repeated choices: households’ choices of appliance efficiency level. Review of Economics and Statistics 80: 647–657.

[13] Bastin F, Cirillo C, Toint PL (2006) Application of an adaptive Monte-Carlo algorithm for mixed logit estimation. Transportation Research part B 40: 577–593.

[14] Hammersley JM (1960) Monte Carlo methods for solving multivariable problems. Annals of the New York Academy of Sciences 86: 844–874.

[15] Halton JH (1960) On the efficiency of certain quasi-random sequences of points in evaluating multi-dimensional integrals. Numerische Mathematik 2: 84–90.

[16] Train K (1999) Halton sequences for mixed logit. Working paper, Department of Economics, University of California, Berkeley.

[17] Bhat CR (2001) Quasi-random maximum simulated likelihood estimation of the Mixed MultiNomial Logit model. Transportation Research Part B 35: 677–693.

[18] Bhat CR (2003) Simulation estimation of mixed discrete choice models using randomized and scrambled Halton sequences. Transportation Research part B 37: 837–855.

[19] Morokoff WJ, Caflisch RE (1995) Quasi-Monte Carlo Integration. Journal of Computational Physics 122: 218–230.

[20] Chiou L, Walker JL (2007) Masking identification of discrete choice models under simulation methods. Journal of Econometrics 141: 683–703.

[21] Walker JL (2001) Extended Discrete Choice Models: Integrated Framework, Flexible Error Structures, and Latent Variables. Ph.D. dissertation, Massachusetts Institute of Technology.

[22] Walker JL, Ben-Akiva M, Bolduc D (2007) Identification of Parameters in Normal Error Component Logit-Mixture (NECLM) Models. Journal of Applied Econometrics 22: 1095–125.

[23] Sándor Z, Train K (2004) Quasi-random simulation of discrete choice models. Transportation Research part B 38: 313–327.

[24] Sivakumar A, Bhat CR, Ökten G (2005) Simulation estimation of mixed discrete choice models with the use of randomized quasi-monte carlo sequences: A comparative study. Transportation Research Record 1921: 112–122.

[25] Munger D, L’Ecuyer D, Bastin F, Cirillo C, Tuffin B (2012) Estimation strategies for complex discrete choice models. Transportation Research part B 46: 305–320.

[26] Greene WH (1997) Econometric analysis. Third edition. Prentice Hall, Upper Saddle River, New Jersey, USA.

[27] Train K (2009) Discrete Choice Methods with Simulation. Cambridge University Press.

[28] Andersen LM (2008) Antithetic Halton draws, in Information Provision to Consumers as an Instrument of Environmental Regulation, PhD Series No. 130 −2008, Department of Economics, University of Copenhagen.

[29] Andersen LM (2011) Animal Welfare and Eggs – Cheap Talk or Money on the Counter? Journal of Agricultural Economics 62: 565–584.

[30] Cappellari L, Jenkins SP (2006) Calculation of Multivariate Normal Probabilities By Simulation, With Applications to Maximum Simulated Likelihood Estimation. IZA Discussion Paper No.2112. Available: http://ssrn.com/abstract=900829. Accessed 2014 July.

