# Supplementary material for: Obtaining Reliable Likelihood Ratio Tests from Simulated Likelihood Functions
Source: PLoS One. 2014 Oct 20;9(10):e106136. doi: 10.1371/journal.pone.0106136 (PMC4203670; doi:10.1371/journal.pone.0106136)
Supplement: Appendix S1 — (DOC) [file pone.0106136.s001.doc]

# S1: Simulated data

The number of individuals is 1,000 and each individual makes 20 choices. The number of alternatives is 4.

The simulated utility of the four alternatives is defined as:

$\begin{matrix} mean\left[\begin{matrix} u_{A} \\ u_{B} \\ u_{C} \\ u_{D} \end{matrix}\right]=\left[\begin{matrix} 0 \\ 0.1 \\ 1 \\ 2 \end{matrix}\right], & std.dev\left[\begin{matrix} u_{A} \\ u_{B} \\ u_{C} \\ u_{D} \end{matrix}\right]=\left[\begin{matrix} 0 \\ 1 \\ 0.1 \\ 3 \end{matrix}\right], & corr\left[\begin{matrix} u_{A} \\ u_{B} \\ u_{C} \\ u_{D} \end{matrix}\right]=\left[\begin{matrix} 0 & 0 & 0 & 0 \\ & 1 & 0 & 0 \\ & & 1 & 0 \\ & & & 1 \end{matrix}\right] \end{matrix}$ S

which means that the simulated covariance is defined as:

$\mathrm{cov}\left[\begin{matrix} u_{A} \\ u_{B} \\ u_{C} \\ u_{D} \end{matrix}\right]=\left[\begin{matrix} 0 & 0 & 0 & 0 \\ & 1 & 0 & 0 \\ & & 0.01 & 0 \\ & & & 9 \end{matrix}\right]$ S

Alternative A is used as the base. The mean and the standard error of the base alternative is zero and the realization of the utility is:

$\begin{matrix} mean\left[\begin{matrix} u_{B} \\ u_{C} \\ u_{D} \end{matrix}\right]=\left[\begin{matrix} 0.0834 \\ 0.9981 \\ 2.0682 \end{matrix}\right], & \mathrm{cov}\left[\begin{matrix} u_{B} \\ u_{C} \\ u_{D} \end{matrix}\right]=\left[\begin{matrix} 0.9868 & 0.0021 & -0.0633 \\ & 0.0097 & \text{-0.0004} \\ & & 9.2070 \end{matrix}\right] \end{matrix}$ S

The values in S(3) are used as the *true* parameter values in the paper.
